# *CTCF* variant begets to short stature by down-regulation of IGF1

**DOI:** 10.1530/JME-22-0193

**Published:** 2023-04-06

**Authors:** Hong Chen, Weiyu Li, Suping Zhang, Yunteng Sun, Yiping Shen, Ruimin Chen

**Affiliations:** 1 Department of Endocrinology, Genetics and Metabolism, Fuzhou Children’s Hospital of Fujian Medical University, Fuzhou, China; 2 Department of Genetic and Metabolic Central Laboratory, the Maternal and Child Health Care Hospital of Guangxi Zhuang Autonomous Region, Guangxi Birth Defects Prevention and Control Institute, Nanning, China; 3 Division of Genetics and Genomics, Boston Children's Hospital, Harvard Medical School, Boston, Massachusetts, USA

**Keywords:** *CTCF*, gene, IGF1, rhGH, short stature

## Abstract

Pathogenic variants in the transcription factor CCCTC-binding factor (*CTCF*) are associated with mental retardation, autosomal dominant 21 (MRD21, MIM#615502). Current studies supported the strong relationship between *CTCF* variants and growth, yet the mechanism of *CTCF* mutation leading to short stature is not known. Clinical information, treatment regimens, and follow-up outcomes of a patient with MRD21 were collected. The possible pathogenic mechanisms of *CTCF* variants leading to short stature were investigated using immortalized lymphocyte cell lines (LCLs), HEK-293T, and immortalized normal human liver cell lines (LO2). This patient received long-term treatment with recombinant human growth hormone (rhGH) which resulted in an increased height of 1.0 SDS. She had low serum insulin-like growth factor 1 (IGF1) before the treatment and the IGF1 level was not significantly increased during the treatment (−1.38 ± 0.61 SDS). The finding suggested that the *CTCF* ^R567W^ variant could have impaired IGF1 production pathway. We further demonstrated that the mutant CTCF had a reduced ability to bind to the promoter region of IGF1, consequently significantly reducing the transcriptional activation and expression of IGF1. Our novel results demonstrated a direct positive regulation of CTCF on the transcription of the *IGF1* promoter. The impaired IGF1 expression due to CTCF mutation may explain the substandard effect of rhGH treatment on MRD21 patients. This study provided novel insights into the molecular basis of CTCF-associated disorder.

## Introduction

CCCTC-binding factor (coded by *CTCF* gene, OMIM *604167), as a transcription insulation protein, plays a key role in regulating the temporal and spatial transcription of genes related to growth in mammals and topologically associated chromatin loop formation (Lupiáñez *et al.* 2015, Liu *et al.* 2019). Pathogenic variants in the *CTCF* gene are associated with mental retardation, autosomal dominant 21 (MRD21, MIM #615502) with short stature, mild facial deformities, and mental retardation (Konrad *et al.* 2019). Since the initial report by Gregor *et al.* in 2013, more than 50 intragenic pathogenic *CTCF* variants have been reported, providing sufficient evidence to establish the strong relationship between *CTCF* variants and MRD21 per ClinGen gene curation classification (https://www.clinicalgenome.org/) (Gregor *et al.* 2013, Chen *et al.* 2019, Konrad *et al.* 2019). The dosage alteration of the *CTCF* variants could be one of the mechanisms for cognition impairment (Gregor *et al.* 2013, Konrad *et al.* 2019). Patients with MRD21 consistently have short stature, yet how CTCF variants lead to short stature is ill-defined.

We performed mechanistic analyses, revealed impaired insulin-like growth factor 1 (IGF1) expression in a patient with *CTCF* pathogenic variant, and uncovered decreased binding of mutant CTCF to the promoter of the *IGF1* gene in cell models. Our study implicates that the CTCF variant by affecting IGF1 expression may be one of the reasons for short stature in patients with MRD21.

## Materials and methods

### Study subjects

This study was reviewed and approved by the Ethics Committee of Fuzhou Children's Hospital of Fujian Medical University (No. 2019-33) and was conducted in agreement with the Declaration of Helsinki. Written and informed consent was obtained from the parents of the proband.

### Clinical evaluations

The parents signed informed consent for the recombinant human growth hormone (rhGH) treatment. Clinical follow-up information including height, weight, growth hormone treatment, insulin-like growth factor 1 (IGF1), insulin-like growth factor binding protein 3 (IGFBP3), and bone age was collected from birth to 12.5 years old. Height and weight were plotted on the standardized growth charts for Chinese children and adolescents aged 2–18 years (Li *et al.* 2009).

### Molecular modeling

The three-dimensional structure of human CTCF protein was modeled on the crystal structure (PDB entry 5YEL) (https://www.uniprot.org/). Bioinformatic tools from HOPE (https://www3.cmbi.umcn.nl/hope/) were used to model and predict the effects of the p.R567W variant on CTCF. The interactions between amino acid residues were analyzed using DynaMut (http://biosig.unimelb.edu.au/dynamut/) (Rodrigues *et al.* 2018). In addition, we predicted the effect of mutations on protein–nucleic acid interactions by the webserver mCSM-NA (http://structure.bioc.cam.ac.uk/mcsm_na). Finally, PyMOL 2.5 was used for the visualization of the docking results.

### Immortalized lymphoblastic cell lines construction

A standard procedure was adopted for obtaining peripheral blood mononuclear cells (PBMCs) from the patient and healthy donors via leukaphereses. The PBMC was separated from the blood by density gradient centrifugation (Küry *et al.* 2017). The EBV (Epstein-Barr virus) B95-8 virus was produced from B95-8 cells with conditional expression of ZTA (Wang *et al.* 2019). Cells were cultured at a density of 1000 cells/mL in RPMI1640/10% fetal bovine serum for 10 days. The virus-containing supernatant was collected and filtered through a 0.45 μm filter to remove cells and cell debris. The immortalized lymphoblastic cell lines (LCLs) were constructed through the co-cultivate of peripheral monocytes and EBV with the drug cyclosporin A (CYA) (Solabio, Burlington, MA, USA) and phytohemagglutinin (PHA) (Sigma-Aldrich) (Dikalov *et al.* 2007).

### Wildtype and mutant CTCF gene expression plasmid construction

Wildtype full-length human* CTCF* complementary DNA (NM_ 006565.3) was chemically synthesized and cloned into the plasmid vector pCMV-MCS-FLAG. The c.1699C>T variant was introduced into the *CTCF* sequence using ClonExpress Ⅱ One Step Cloning Kit (Vazyme, Nanjing, China). The cDNA was amplified using 2× Phanta Max Master Mix (Vazyme). The connected product was transformed into *Escherichia coli* DH5a. Positive clones were selected and sequenced to verify the correct insertion sequence. All operations were following the manufacturer's requirements. The primer sequences were designed by the CE Design of the Vazyme official website (Supplementary Table 1, see section on supplementary materials given at the end of this article) and synthesized by Sangon Biotech.

### Cell culture and transfection

Cell culture media were prepared and maintained according to standard cell culture procedures. The HEK-293T (ATCC® CRL-11268) were cultured in high-glucose DMEM (Hyclone®), containing 10% fetal bovine serum and 1% penicillin-streptomycin at 37°C with 5% CO_2_. B958-EBV, LCLs, and immortalized normal human liver cell line LO2 cells were cultured with RPMI1640 medium with 10% fetal bovine serum at 37°C with 5% CO_2_. HEK-293T cells were cultured in 24-well plates. For transient transfections, 70% confluent HEK-293T cells were transfected with 500 ng plasmid DNA per well by Hieff Trans™ Liposomal Transfection Reagent (Cat#40802, Yeasen, Shanghai, China). All cell lines were routinely tested for mycoplasma contamination.

For transient transfection, HEK-293T and LO2 cells were seeded in a 6-well plate until 60–80% confluence. The volume/mass ratio of Exfect2000 Transfection Reagent (Vazyme) to DNA was 3:1. Transfection efficiency was evaluated using quantitative real-time polymerase chain reaction (qPCR) and Western blot. After transfection for 48 h, cells/supernatant was collected for subsequent studies.

### Quantitative real-time polymerase chain reaction

Three groups were tested, including a negative control with only plasmid pCMV-MCS-FLAG, WT with pCMV-MCS-FLAG-WT-CTCF, and experimental groups with pCMV-MCS-FLAG-MUT- CTCF of c.1699C>T. To determine the mRNA level under the impact of the CTCF^R567W^ variant, we performed qPCR assays in three groups in three cell lines, including HEK-293T, LO2, and LCLs. Total RNA was obtained using an easy Isolation Reagent (Vazyme). cDNA was obtained using the HiScript Ⅲ 1st Strand cDNA Synthesis Kit (Vazyme). Primers were designed by NCBI-Primer Blast (National Center for Biotechnology Information (NCBI)) (Supplementary Table 1) and synthesized by Sangon Biotech. Messenger RNA (mRNA) expression levels were quantified by Applied Biosystems StepOnePlus Real-Time PCR System using 2×ChamQ Universal SYBR qPCR Master Mix (Vazyme). At least three independent experiments were conducted. Data were expressed as mean ± s.d. and analyzed using *t*-test.

### Western blot analysis

To analyze the protein level of the CTCF, we performed Western blot assays in three groups in the LO2 cell line using two distinct rabbit monoclonal primary antibodies: anti-CTCF antibody (Abcam) and anti-FLAG antibody (Abcam), the latter being a marker sequence of the target gene. The cells were collected and lysed in RIPA lysis buffer (Beyotime, Shanghai, China). Equal amount of protein (30 μg), as quantitated using the BCA Protein Assay, from each group was resolved in 8% SDS-PAGE and then electro-transferred onto a polyvinylidene difluoride membranes for western blot analysis (Li *et al.* 2018).

### Enzyme-linked immunosorbent assay

To determine the protein level of IGF1 and IGFBP3, snzyme-linked immunosorbent assays (ELISA) in three groups in three different cell lines were used. The protein expression levels of IGF1 and IGFBP3 were quantified using Human IGF1 ELISA Kit (Elabscience, Wuhan, China) and Human IGFBP-3 ELISA Kit (Elabscience), respectively.

### Luciferase assay

Luciferase assays in HEK-293T cells were used to assess transcription activation. The promoter of *IGF1* (−900 bp to +137 bp) was amplified and inserted into the plasmid vector pGL4.1. HEK-293T cells (1 × 10^4^ cells/well) were seeded in 24-well plates until 60–80% confluence, then *IGF1* reporter vectors were co-transfected with recombinant pGL4.1 luciferase reporter plasmid and wildtype or variant *CTCF* expression vectors with the DNA ratio 1:1 (2.5 μg each) by Exfect2000 Transfection Reagent (Vazyme). Luciferase was evaluated at 24, 36, 48, 60, and 72 h after transfection using the luciferase assay system (Promega).

### Chromatin immunoprecipitation-qPCR

To study the occupation rate of CTCF on the primer region, we performed a chromatin immunoprecipitation (CHIP)-qPCR assay in HEK-293T cells. The cells (1 × 10^4^ cells/well) were seeded in 24-well plates and transfected with the wildtype or variant CTCF expression vectors until 60–80% confluence. Cells were collected 72 h after transfection. The protocol for ChIP-qPCR analysis was performed per MAGnify™ Chromatin Immunoprecipitation System (Invitrogen). The DNA fragments of 500–1000 bp were sonicated and purified. The input was used as the positive control and rabbit IgG served as the negative control. The qPCR analysis followed was performed as steps mentioned earlier. The sequences of primers for the promoter region used in this study are shown in Supplementary Table 1.

### Statistical analysis

Western blotting images were processed using Fiji/Image J software (https://imagej.net/Fiji). Statistical analyses were performed in Graph Pad Prism 8 program. Two-sided *t*-tests were performed to compare groups. At least three independent experiments were conducted to verify the results. Data were considered statistically significant when *P* < 0.05. Data are means ± s.e.m. of three independent experiments. (*P < 0.05; **P < 0.01; ***P < 0.001).

## Results

### Clinical evaluations

**Figure 1:**
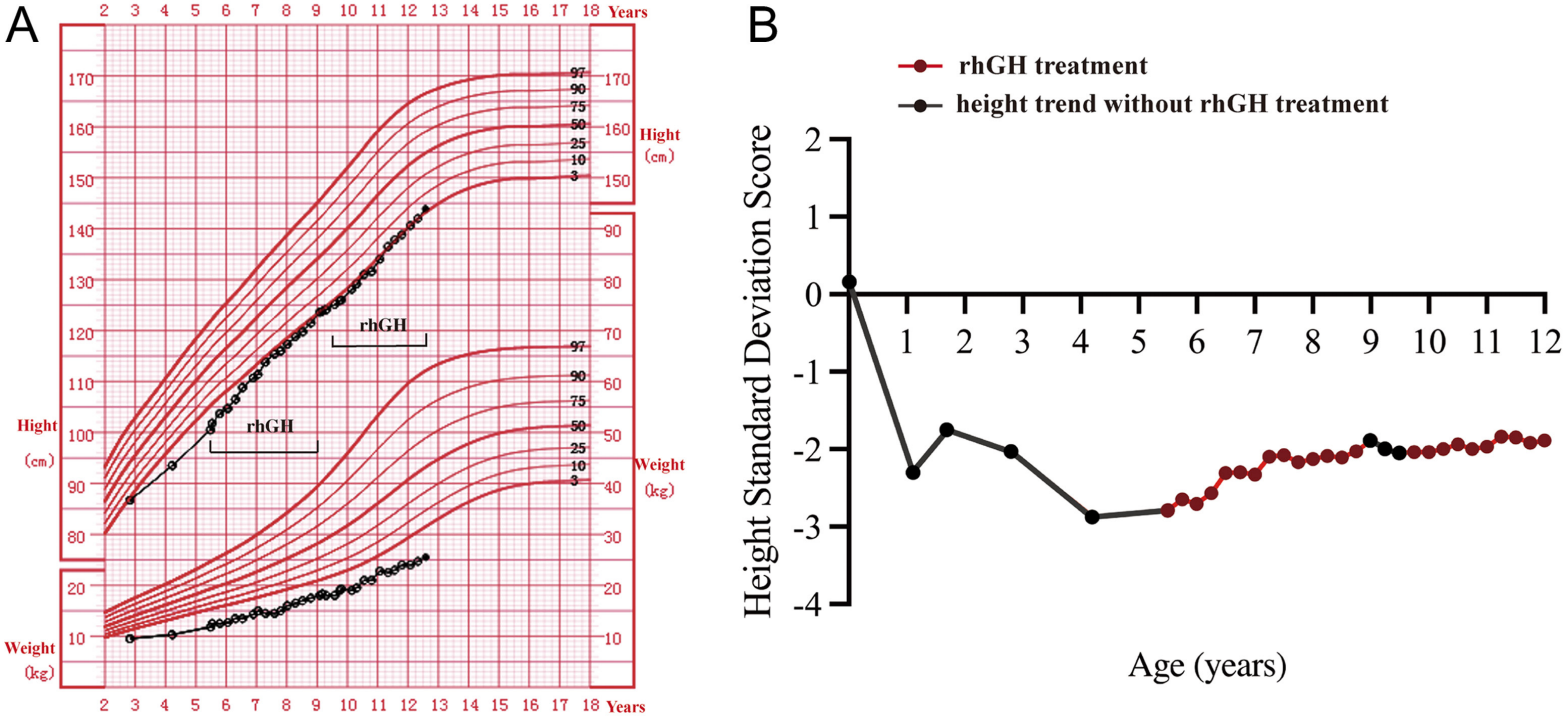
Growth chart of the patient with heterozygous CTCF variant before and during the rhGH treatment. (A) The height and weight curves of the patient from birth to age 12. The black dots represent points of measurement. (B) The height trends during rhGH treatment. A full color version of this figure is available at https://doi.org/10.1530/JME-22-0193.

The patient characteristics, including diagnoses and detailed clinical features, are described in our previous paper (Chen *et al.* 2019). In this report, we further detail the postnatal growth and development as well as the treatment and follow-up of the child carrying the *CTCF* gene variant (c.1699C>T, p.R567W). Her birth height was normal. In the first years of life, linear growth was poor, dropping to a height of −2.87 SDS at the age of 5. Due to her short stature, she received rhGH therapy (0.10–0.18 IU/kg/day) from 5.5 to 12.5 years of age. During 6.5 years of rhGH treatment, her height increased from −2.79 SDS to −1.77 SDS (Table 1). In the first year of treatment, the height increased by 0.48 SDS (Fig. 1A and B). Notably, she had low IGF1 levels before treatment and they did not increase significantly during rhGH treatment (−1.38 ± 0.61 SDS). IGFBP3 levels were normal. In addition, she had poor weight gain after birth until the latest follow-up (−2.44 ± 0.57 SDS). She entered puberty spontaneously at 9.5 years but has no menarche at 12.5 years. Details of follow-up, treatment, and outcome treatment are provided in Table 1.

**Table 1:** Genetic information and response to growth hormone therapy in the patient.

Age (years)	Duration of GH treatment (years)	Dose of rhGH (IU/kg/d)	Height (cm)	HSDS	Weight (kg)	WSDS	Bone age (years)	IGF1 (pg/mL)	IGF1 (SDS)	IGFBP3 (mg/L)
Birth	0	0	50.0	0.16	2.65	−2.13	ND	ND	ND	ND
1.1	0	0	70	−2.3	7.5	−0.73	ND	<25.0	<−2.0	ND
1.7	0	0	78.4	−1.75	9.0	−1.39	ND	46.0	−1.5	ND
2.8	0	0	86.7	−2.03	9.5	−2.91	ND	ND	ND	ND
4.2	0	0	93.5	−2.87	10.3	−2.2	2.0	ND	ND	ND
5.5	0	0.10	101.8	−2.79	12.5	−2.97	3.0	92.0	−1.0	ND
6.0	0.5	0.12	104.7	−2.71	12.7	−2.23	ND	112.0	−0.97	3.30
6.5	1	0.12	108.8	−2.31	13.5	−2.35	ND	122	−0.88	3.33
7.0	1.5	0.12	111.4	−2.33	15.0	−2.77	ND	148	−0.65	ND
7.5	2.0	0.12	115.4	−2.08	14.5	−2.01	5.9	98.8	−1.71	3.6
8.0	2.5	01.3	117.3	−2.13	16	−2.88	6.3	110	−1.78	2.71
8.5	3.0	0.15	119.8	−2.11	17.0	−2.84	7	121	−1.76	3.12
9.0	3.5	0.16	123.6	−1.89	18.0	−2.88	7.5	122	−2.10	4.18
9.5	3.5	0	125.1	−2.05	18	−2.83	8	107	−2.26	3.76
10.0	4.0	0.16	128.1	−2.04	19	−2.93	ND	249	−0.80	2.12
10.5	4.5	0.17	131	−1.94	21	−2.40	8.5	208	−1.20	4.68
11.0	5.0	0.17	134	−1.97	22.8	−2.03	ND	317	−0.17	4.83
11.5	5.5	0.17	137.8	−1.85	23	−2.65	9.5	256	−0.95	ND
12.0	6.0	0.18	140.6	−1.89	24	−2.71	9.8	170	−2.1	4.29
12.5	6.50	0.18	143.9	−1.77	25.5	−2.69	ND	170	−2.2	4.67

GH, growth hormone; HSDS, height standard deviation score; IGF1, insulin-like growth factor 1; IGFBP3, insulin-like growth factor binding protein 3; ND, not done; rhGH, recombinant human growth hormone; WSDS, weight standard deviation score.

### Three-dimensional structure modeling of the CTCF^R567W^ variant

**Figure 2:**
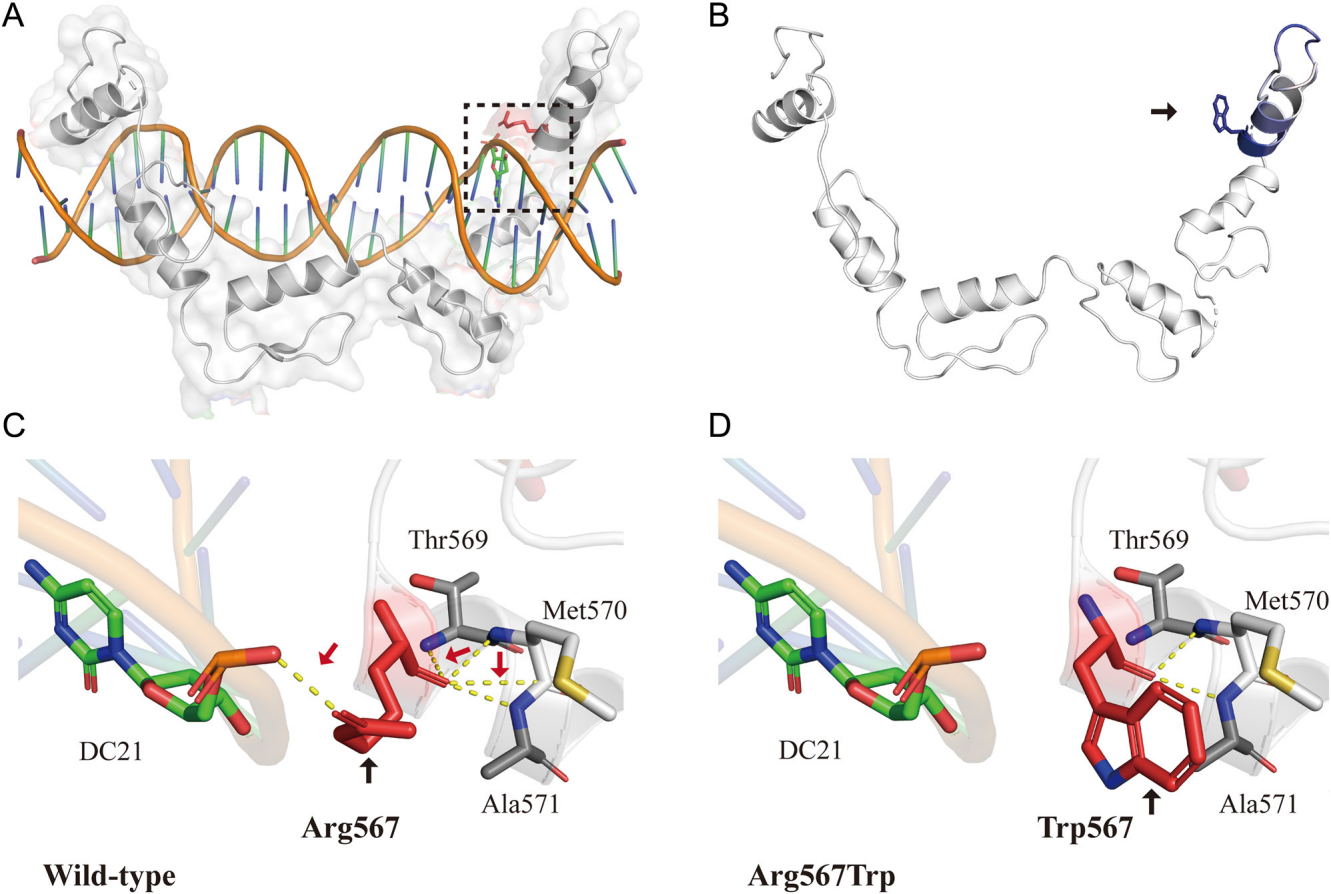
Three-dimensional structure modeling of the CTCFR567W variant. (A) Three-dimensional structure of wildtype CTCF protein. The crystal structures above were based on the UniProt reference molecular structure (PDB: 5YEL). The black dotted box marks the position of the Arg567 variant. The amino acid Arg567 was labeled red. (B) Δ Vibrational entropy energy | Visual representation of variant: amino acids colored based on the vibrational entropy change of the variant. Blue represents a rigidification of structure. (C, D) Interaction prediction between amino acid residues: wildtype and variant residues are represented as red sticks. These are placed alongside surrounding residues which are also involved in other types of interactions. Red arrows indicate interactions between altered amino acids. Black arrows indicate wildtype or variant amino acid residues. Hydrogen bonding is shown with a yellow dotted line. DC, cytosine deoxyribonucleoside. A full color version of this figure is available at https://doi.org/10.1530/JME-22-0193.

PyMol software was used to visualize the 3D structure of the wildtype of CTCF (amino acids: 450–580) (Fig. 2A). This is then used as a basis for further modeling of protein structure to predict possible changes in protein flexibility after mutation. The p.R567W variant reduced the local flexibility of the protein (ΔΔSVibENCoM: −4.186 kcal/mol/K) (Fig. 2B). In addition, the mCSM-NA software foretells that the p.Arg567Trp variant reduces the affinity of the Trp567 residue to DNA (ΔΔG - 0.405 kcal/mol).

Lastly, the HOPE and DynaMut online servers were used to predict the local interactions between wildtype amino acid residues and variant amino acid residues. As a result of the point mutation, arginine (Arg/R) was substituted with tryptophan (Trp/W) resulting in significant differences in residue size. Moreover, the variant introduced a more hydrophobic residue that could disrupt the folding of proteins and/or cause hydrogen bond loss (Fig. 2C and D). The differences in amino acid properties disturb a Zinc-finger domain which may disrupt its binding to DNA. The special structural disturbance of the p.Arg567Trp variant cancels the local hydrogen bond pattern of the Arg567Trp residue to the adjacent cytosine deoxyribonucleoside 21 (DC21) residue, thereby attenuating the flexibility of the variant.

### CTCF^R567W^ variant inhibited the expression of *IGF1* gene

**Figure 3:**
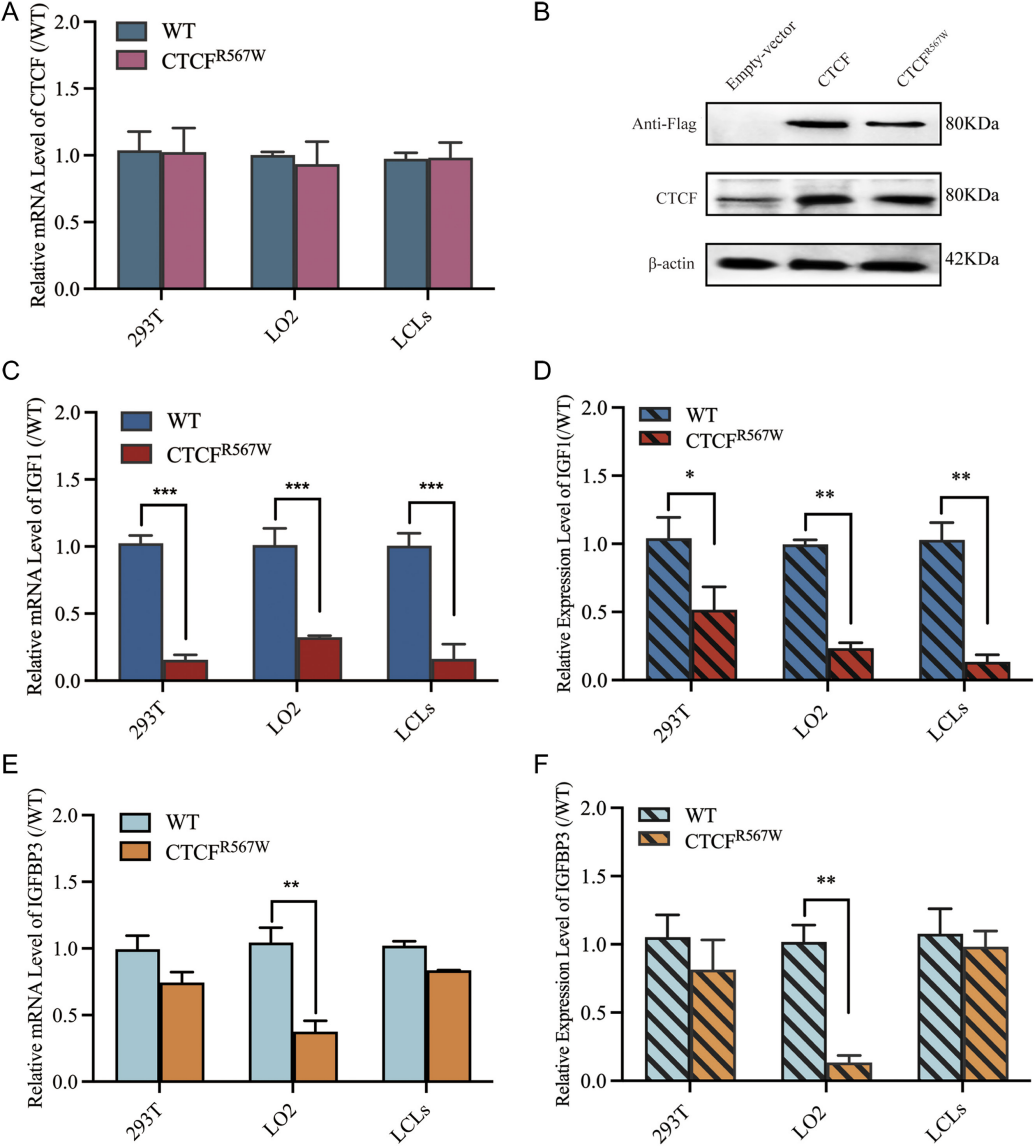
The CTCF^R567W^ variant inhibition of IGFs gene expression. (A) qPCR analysis of the expression level of *CTCF* mRNA in the wild and mutant groups in three different cell lines. (B) Western blot analysis of the expression level of CTCF protein in the wildtype and mutant groups in LO2 cells. FLAG is a label protein of vectors. (C) In HEK-293T, LO2, and LCLs, mRNA levels of *IGF1* were measured using quantitative PCR. (D) IGF1 protein levels were analyzed with ELISA. (E) In HEK-293T, LO2, and LCLs, mRNA levels of IGFBP3 were measured using quantitative PCR. (D) IGFBP3 protein levels were analyzed with ELISA. For quantitative PCR, results were normalized to β-actin as a reference and compared with control cells transfected with an empty vector plasmid (*n* ≥ 3; mean ± s.e.m. shown; ***P < 0.001 by Student *t*-test). For Western blotting, results were normalized to β-actin as a reference (*n* = 3; mean ± s.e.m. shown; *P < 0.05, **P < 0.01, and ***P < 0.001). A full color version of this figure is available at https://doi.org/10.1530/JME-22-0193.

IGF1 is a key regulator of growth, differentiation, and metabolism of bone cells (Missailidis *et al.* 2020). To further assess the effect of the CTCF^R567W^ variant on IGF1 signaling pathways, we analyzed the effects of the variant on the expression of key genes for linear growth. We overexpressed the wild-type or mutant *CTCF* gene in the HEK-293T cells and immortalized normal human liver cell line LO2. Our analysis showed that there was no significant difference in CTCF mRNA expression and protein levels between the wildtype and mutant groups (Fig. 3A and B). We then assessed the mRNA and protein levels of IGF1 and IGFBP3 genes in the HEK-293T, LO2, and LCLs. In HEK-293T, LO2, and LCLs, *IGF1* mRNA expression in the mutant group was 15.5, 32.4, and 16.2% of that in the wildtype group, respectively (Fig. 3C), while the protein expression had decreased to 41.5, 23.4, and 13.4% of that in the wildtype (Fig. 3D). In LO2 cells, the mRNA expression of IGFBP3 in the mutant group was 36.0% to that in the wildtype group (Fig. 3E), and the protein expression decreased to 42.7% of the wildtype (Fig. 3F). However, in HEK-293T and LCLs, CTCFR567W variant did not affect the expression of IGFBP3. These results demonstrate that the CTCF^R567W^ variant inhibits the expression of the *IGFBP3* gene in LO2 cells and down-regulated the expression of the *IGF1* gene in all three different cell lines.

### CTCF^R567W^ variant directly decreases the transcription of *IGF1* gene

**Figure 4:**
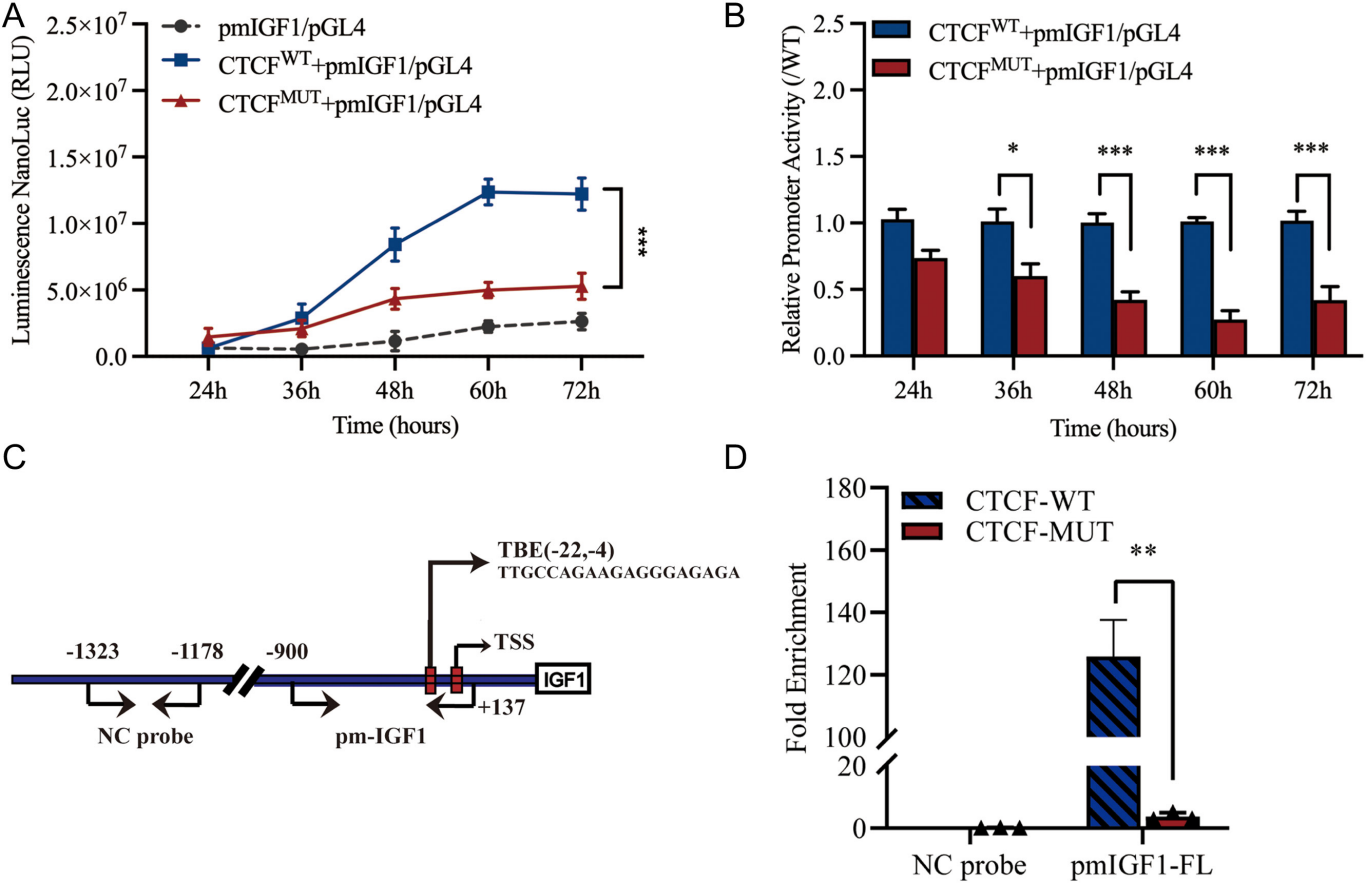
Regulation of *IGF1* expression by CTCF. (A, B) The transcriptional activity of *IGF1* responds to the CTCF^R567W^ variant by luciferase assay in HEK-293T cell. Co-transfected HEK-293T cells with wildtype or mutant CTCF expression plasmids and *IGF1* reporter recombinant plasmids. (A) Luciferase activity was determined at 24, 36, 48, 60, and 72 h after transfection. (B) For comparison, the wildtype values were adjusted to 1’; a histogram represents the relative value of transcriptional activities. Data are means ± s.e.m. of three independent experiments (*P < 0.5; **P < 0.001; ***P < 0.0001; *t*-test). (C, D) The interaction between the CTCF^R567W^ variant and *IGF1* by CHIP-qPCR assay. (C) The diagram of full-length (FL) of *IGF1* gene promoter (pmIGF1-FL). (D) CHIP-qPCR assay of the occupation rate of CTCF on the *IGF1* primer region. NC, negative control; pm: promoter; TBE:,transcription binding elements; TSS, transcription start sites. Data are means ± s.e.m. of three independent experiments (*P < 0.5; **P < 0.001; ***P < 0.0001; *t*-test). A full color version of this figure is available at https://doi.org/10.1530/JME-22-0193.

To confirm whether CTCF^567W^ variant directly regulates the transcriptional activation of *IGF1*, we performed a luciferase reporter gene assay in HEK-293T cells. The change of the measured value for 72 consecutive hours was shown as the broken lines (Fig. 4A). Relative luminescence units (RLU) data represent the fold change in the mutant group with the wild group set at a mean value of 1 (Fig. 4B). The results of RLU demonstrated the activation of the *IGF1* promoter reporter vector in the mutant group was robust and significantly down-regulated and, at 60 h after transfection, the relative luciferase activity in the mutant group reached a nadir, reduced by 72.56% compared to the wildtype group (Fig. 4A and B).

### Reduced binding of CTCF^R567W^ on *IGF1* gene

To further investigate the interactions between CTCF and the promoter regions of *IGF1* gene in the nucleus, chromosome immunoprecipitation followed by qPCR (CHIP-qPCR) in HEK-293T cell lines was performed. JASPAR online website predicted that CTCF has one binding site in the* IGF1* promoter region (Fig. 4C). Wildtype CTCF protein is highly enriched in this region and promotes transcription of *IGF1*. Compared with the wildtype, the CTCF^R567W^ variant was significantly less enriched in the full length of the *IGF1* promoter, only 3% of the wildtype (Fig. 4D).

## Discussion

Heterozygous mutations in the *CTCF* gene are associated with short stature (Chen *et al.* 2019). To date, there are no reports of patients with MRD21 due to mutations treated with rhGH. This patient received long-term treatment with rhGH (6.5 years) and improved in height by 1.0 SDS. Although there are no well-established criteria for rhGH effectiveness, a recent study reported a mean height increase of 2 SDS after 6 years of rhGH treatment in idiopathic short stature (ISS) patients (Zhao *et al.* 2021). Our patient demonstrated a marginal degree of improvement in growth velocity with rhGH therapy, but not as significant as the growth effect on ISS patients (Ying *et al.* 2018). The increase in IGF1 concentrations during rhGH treatment is a surrogate marker reflecting the efficacy of treatment (Ying *et al.* 2018). Previous studies have shown that in patients with IGF1 deficiency, an improved growth response is possible if a higher dose of rhGH is prescribed (Walenkamp *et al.* 2013). Yet, our patient showed no increase in IGF1 levels during rhGH treatment, even though the dose of rhGH was increased from 1.0 to 1.8 IU/kg/day. Therefore, we propose that mutant CTCF impacts IGF1 gene expression, which in turn leads to short stature and the non-ideal response of long-term rhGH treatment.

The CTCF protein is encoded by the *CTCF* gene located at chromosome 16q22.1 and generates a highly conserved transcription factor of approximately 80 KDa (Soshnikova *et al.* 2010). The CTCF protein consists of 11 domains of C2H2-type zinc finger structures which bind over 30,000 DNA motifs with different combinations numerous in the human genome (Jerković *et al.* 2017). CTCF is involved in many gene expression regulating processes, including transcription regulation, imprinting, RNA shearing, chromosome loop formation, and X-inactivation (Franke *et al.* 2016, Hansen *et al.* 2017, Lazniewski *et al.* 2019, Saldaña-Meyer *et al.* 2019). It plays a vital role in regulating developmental genes and governs vertebral growth and differentiation (Lupiáñez *et al.* 2015, Liu *et al.* 2019). Based on the available clinical evidence, *CTCF* pathogenetic variants have been implicated in human short stature, but the pathogenesis is unexplained. In the present study, we found the Arg567Trp residue of CTCF is located in the α-helix of the 11th zinc finger domain and is evolutionarily highly conserved in vertebrates. The results of the DynaMut online server predicted that the p.Arg567Trp variant would be neutral, which infers that the overall structure of CTCF^R567W^ is stable. Based on the 3D crystal structure of human CTCF (PDB: 5yel), the results indicate that the substitution of Arg with the natural Trp in the hydrogen core destabilizes the α-helix of the zinc finger structure by canceling the interaction between Arg567 and DC21. These results are in agreement with those of a previous study (Gregor *et al.* 2013). The flexibility of the α-helix is an indispensable mechanism for ensuring protein function, protein–protein interactions, and protein–substrate binding (Jarosch 2005, Pauwels & Tompa 2016). Thus, the impaired flexibility of the α-helix and affinity with the deoxynucleotide of the CTCF^R567W^ may account for the disrupted function of the CTCF.

Previous studies have confirmed the importance of IGF1 in fetal growth in utero and after birth (Netchine *et al.* 2011, Shcheglovitov *et al.* 2013). Most studies emphasize its transcriptional function in folding mammalian genomes into spatial domains to block or intensify the effect of enhancers on promoters. On the other hand, several studies conclude that CTCF also interacts with the promoter region to activate the transcription directly, independent of the formation of chromosome loops. Our data suggest that the CTCF^R567W^ variant is responsible for reduced levels of IGF1, and this variant could potentially affect other short stature-related genes in cells. We compared the IGF1 levels between cells over-expressed with wildtype CTCF or mutant CFCT: the significant changes in IGF1 level could be best explained by the variant CTCF. Following that, we performed qPCR and ELISA experiments with patient-derived immortalized lymphocytes (heterozygotes) and obtained similar results as in the overexpressed cell lines. This effect could be due to a dominant negative mechanism. Compared with the wild group, the CTCF^R567W^ variant had a reduced ability to bind DNA. Furthermore, CHIP-qPCR showed that CTCF interacted with the *IGF1* promoter. The CTCF^R567W^ variant significantly reduced its interaction with the *IGF1* promoter sequence and inhibited its transcriptional activation of the *IGF1* gene. The direct regulation of the *IGF1* gene promoter by CTCF may explain the short stature and poor response to rhGH in the MRD21 patient. Insofar as CTCF can bind to and regulate many genes, our experiments suggest that the CTCF variant may influence human height through impaired IGF1 production. And, as for now, the coupling between CTCF and human height is a conjecture.

## Conclusions

In sum, our findings reveal that the CTCF^R567W^ variant, located in the α-helix of the 11^th^ zinc finger, significantly hinders the DNA binding of the CTCF^R567W^ to *IGF1* gene promoter, thereby down-regulating *IGF1* expression and signaling. The low IGF1 level in patient and the reduced expression of IGF1 due to CTCF mutation could partially explain the short stature phenotype and the unsatisfactory height improvement of the patient with long-term rhGH treatment. Taken together, our study provides novel insights into the molecular mechanisms by which *CTCF* gene variants contribute to short stature. Studies on additional patients and variants could further corroborate our findings.

## Supplementary Material

Supplementary Table 1. Primer list

## Declaration of interest

There are no competing financial or nonfinancial interests.

## Funding

This study was supported by the Fuzhou Science and Technology Plan Project (2019-S-80), Fuzhou Science and Technology Project (2021-S-197), and Health and Health Science and Technology Program Projects of Fujian Province (2021GGA068) and National Natural Science Foundation of China http://dx.doi.org/10.13039/501100001809 grant 82071276.

## Data availability statement

All datasets generated for this study are included in the article.

## Consent for publication

All authors consent for publication.

## Ethics approval and consent to participate

This study was reviewed and approved by the Ethics Committee of Fuzhou Children's Hospital of Fujian Medical University and was conducted in agreement with the Declaration of Helsinki.

## Author contribution statement

Hong Chen: Writing-Original Draft, Investigation, Data curation; Weiyu Li: Writing-Original Draft, Investigation, and Validation; Suping Zhang and Yunteng Sun: Investigation and Validation; Yiping Shen; Conceptualization and Writing-Review & Editing; Ruimin Chen: Conceptualization, Methodology, Writing-Review & Editing.
