# Supplementary material for: CTCF variant begets to short stature by down-regulation of IGF1
Source: J Mol Endocrinol. 2023 Apr 6;70(4):e220193. doi: 10.1530/JME-22-0193 (PMC10160550; doi:10.1530/JME-22-0193)
Supplement: Supplementary Table 1. Primer list [file supplementary_table_1.pdf]

Supplementary Table 1. Primer list

Usage	Gene	Gene ID	NCBI Reference Sequence	Forward primers (5’→3’)	Reverse primers (3’→5’)
**PCR**	IGF1	4214	NM_001111283.3	AGCTGGCTTGGACCATGTTGC	GCAGGCTCTATCTGCTCTG
	CTCF	10664	NM_006565.4	GAAGGTGATGCAGTCGAAGCC	CCGGTCCATCATGCTGAGG
	CTCF^{R567W}	10664	NM_006565.4	CACGTTGGAATACCATGGCAAGACAT GCTGAT	ATGGTATTCCAGACGTGTAAATGTT TTCCCACACTTA
**qPCR**	IGF1	3479	NM_023067	CTCTTCAGTTCGTGTGTGGAGAC	CAGCCTCCTTAGATCACAGCTC
	IGFBP3	3486	NM_001013398.2	CGCTACAAAGTTGACTACGAGTC	GTCTTCCATTTCTCTACGGCAGG
	CTCF	10664	NM_006565.4	GACCACACAAGTGCCATCTCTG	ATGTCGCAGTCTGGGCACTTGT
